# Parental intentions and requests to provide pain care for their infants in neonatal intensive care units

**DOI:** 10.3389/fped.2024.1512917

**Published:** 2025-01-03

**Authors:** Aya Shimizu, Takeshi Arimitsu, Kana Harada, Mio Ozawa

**Affiliations:** ^1^ Maternal Nursing and Midwifery, Graduate School of Nursing, Osaka Metropolitan University, Habikino, Osaka, Japan; ^2^ Department of Pediatrics, Keio University School of Medicine, Tokyo, Japan; ^3^ Toho University Omori Medical Center, Tokyo, Japan; ^4^ Division of Nursing Science, Graduate School of Biomedical & Health Sciences, Hiroshima University, Hiroshima, Japan

**Keywords:** neonatal intensive care unit, nonpharmacological pain care, parents, preterm infants, family-centered care

## Abstract

**Purpose:**

The Japan Association of Neonatal Nursing evaluated the pain care provided by parents to their infants admitted to the neonatal intensive care unit (NICU). However, further collaborations with families based on family-centered care are necessary to clarify the parental intentions and requests regarding pain care for their infants. This study aimed to describe the experiences and content of nonpharmacological pain care provided by parents to their infants, the intentions and requests of parents regarding each type of recommended pain care (irrespective of whether they had provided pain care at the NICU), and the reasons for their hesitation to implement specific pain management methods.

**Methods:**

A total of 108 parents with NICU-hospitalized infants, including 66 (65.6%) infants with a birth weight of <1,000 g, voluntarily responded to an anonymous self-administered online electronic survey. Sociodemographic and clinical data were quantitatively analyzed.

**Results:**

In our study population, 30.6% (*N* = 33) had provided pain care to their infants, 56.5% (*N* = 61) hoped to provide pain care in the future, and 40.7% (*N* = 44) expected advice for pain care options from healthcare professionals (HCPs). Swaddling, facilitated tucking, and skin-to-skin contact were the most popular options (≥60%). By contrast, the use of sucrose and breastfeeding (both 13.0%), skin-to-skin contact (7%), and use of expressed breast milk and non-nutritive sucking (both 3.7%) were less frequently used due to indifference or doubts, lack of knowledge about pain care, differences between recommended pain care methods and parental values, and pain care methods being inappropriate for the child's condition.

**Conclusions:**

This survey demonstrated that when parents provide pain care for their children in the NICU, they are required to make choices based on the advice and knowledge offered by HCPs, taking into account the diverse values of parents as well as the overall condition of their infant and their breastfeeding status. Therefore, we suggest that HCPs support parents in choosing not only the recommended care but also the most appropriate pain care for the condition of their infant.

## 1 Introduction

Appropriate pain management in preterm infants improves their developmental prognoses but remains a considerable clinical challenge. In newborns, inhibitory interneurons are present in layer II of the spinal dorsal horn, but the transmission from nociceptive C-fibers is weak, and central inhibitory transmission to pain sensation is poor, affecting brain structure and development, as well as neurobehavioral function (1). Although advanced neonatal medical care has improved the short- and long-term outcomes in preterm infants, we should continue implementing strategies to provide effective analgesic relief for repetitive procedural pain and promote healthy growth and development in preterm infants. Following the trend in the US and Europe in the 2000s, a committee of the Japanese Academy of Neonatal Nursing published the first edition of the “Guidelines for Pain Care of Newborns Hospitalized in the NICU” in December 2014 and revised these guidelines in March 2020 (2), which serves now as a standard for clinical care practice in Minds, a database of clinical guidelines operated by the Japan Council for Quality Health Care. The recommendation statement considers the certainty of the evidence extracted through systematic reviews of clinical publications, and the recommendation level is based on the balance of benefits and harms for neonates, usefulness, safety, and feasibility. The guideline recommendations are based on family-centered care and support parents accompanying their children during procedures and participating in pain care. Specifically, environmental adjustment, swaddling, facilitated tucking, non-nutritive sucking, breastfeeding, use of extracted breast milk, skin-to-skin contact, and kangaroo care are strongly recommended as pain management strategies for families intending to be involved in the pain care of their infants.

A 2016 national survey among parents of infants hospitalized in neonatal intensive care units (NICUs) (3) found that mothers wanted to accompany their children during treatment because they recognized their infants' pain and wanted to help relieve their pain and understand their pain experiences and the course of treatment. However, some mothers did not wish to accompany their infants during treatment because they were concerned about the emotional impact the treatment might have on themselves. They trusted the healthcare professionals (HCPs) and left their children in the care of medical staff, prioritizing rules. A 2022 national survey of HCPs in NICUs showed that environmental adjustments, swaddling, facilitated tucking, and non-nutritive sucking, which can be performed by medical professionals instead of family members, were widely used, whereas breastfeeding and skin-to-skin contact, based on the parents' wishes, were less widely used. Pain care in collaboration with the family requires motivating the family members of the pediatric patient to become involved. Therefore, strengthening support for family collaboration is an urgent clinical issue that should be addressed based on the parents' intentions and requests for analgesia for their infants based on Japanese cultural backgrounds.

Nonpharmacological pain management for preterm infants in the NICU is possible through communication and collaboration between HCPs and parents based on the infant's physical condition and family circumstances. Ultimately, this strategy helps reduce parental anxiety. Nurses interviewed in a focus group on pain management reported that parents can obtain more knowledge about pain and have opportunities to participate in care when collaborating with nurses compared to when the nurse takes the lead and the parents are absent or passive (4). Interactions between parents and HCPs affect parental stress management, and knowledge, participation, childcare, satisfaction, and communication are particularly effective in reducing parental stress and anxiety. Compared to the absence of parent- and nurse-controlled analgesia, parent- and nurse-controlled analgesia, in which parents work with HCPs to manage pain using analgesics (5, 6), tended to decrease pain scores of preterm infants and decreased their opioid intake These findings suggest that families can better understand their children's condition through collaboration with HCPs, which can reduce parental anxiety and increase parental care readiness. Although there is no standardization of care based on mental health screening, HCPs currently tailor individualized care plans in dialogue with parents, considering their mental health status and parental involvement in care. However, there is no mention of whether parents intend to commit to pain care after understanding their child's physical condition or their wishes when selecting and implementing pain care.

The Japan Association of Neonatal Nursing has previously evaluated the pain care that parents provide to infants admitted to the NICU (3, 7). However, further collaboration with families based on family-centered care is necessary to clarify the intentions of parents and their requests to be involved in pain care for their infants. Thus, this study aimed to describe the experiences and content of pain care provided by parents to their infants, the intentions and requests of parents regarding each type of recommended pain care (irrespective of whether the parents provided pain care at the NICU), and the reasons for their hesitation. Based on our findings, the next revision of the “Guidelines for Pain Care of Newborns Hospitalized in the NICU” will not only ensure the reliability of updated evidence based on systematic reviews but can also include recommendations that consider parental intentions and requests. Thereby, HCPs can improve operational methods of parental participation in pain management under a family-centered care model.

## 2 Methods

### 2.1 Design

The study involved an open descriptive survey conducted using anonymous self-administered questionnaires via Microsoft Forms, an online electronic survey (8), among parents with contact to family groups whose children had been hospitalized in the NICU.

### 2.2 Operational definition of terms

Pain: We only focused on procedural pain caused by blood sampling to ensure consistency in the setting because the revised “Guidelines for Pain Care of Newborns Hospitalized in the NICU (2020 updated)” recommend measures only for procedural pain.

Recommended pain care for parents: According to the latest guidelines in Japan (2), recommended nonpharmacological methods of pain relief include swaddling, facilitated tucking, non-nutritive sucking, breastfeeding, use of expressed milk, kangaroo care, skin-to-skin contact, and use of sucrose.

### 2.3 Participants

We enrolled parents of children hospitalized in the NICU who were born after January 2015, i.e., after the first edition of the “Guidelines for Pain Care of Newborns Hospitalized in the NICU” had been published in December 2014. In each family, the survey could be answered by both parents, only the mother, or only the father. Participants who had difficulty understanding, reading, and writing in Japanese or were under 18 years of age were excluded.

### 2.4 Data collection

The study was conducted from February to March 2024. The researchers asked the representatives of Japanese facilities registered with the Premature Infant Family Association to recruit participants. The representatives announced the requested description of the survey by emailing it to registered association members through a mailing list. Each parent received the questionnaire from a peer support group. Parents read the requested description and, if they agreed to participate, they accessed the link or two-dimensional code (i.e., a QR code) in the instructions to answer the questionnaire. The principal investigator responded to their inquiries regarding the study.

### 2.5 Questionnaire

The researchers used a questionnaire based on the “Guidelines for Pain Care of Newborns Hospitalized in the NICU (2020 updated)” (2) and previous research (3). The survey profiled the experiences of parents and their infants during hospitalization in the NICU, parental experiences regarding the pain care for their infant, parental hopes and hesitations regarding each recommended pain care method, and the reasons for hesitating to be involved in pain care in the NICU. The researchers informed the participants that they should complete the survey only once. The survey could be completed in approximately 15 min.

Researchers and family members of the Committee of Developing Guidelines for Pain Care of Newborns Hospitalized in the NICU, the usability and technical functionality of the electronic questionnaire, and the appropriateness of the questionnaire examined whether it was easy to understand correctly and share the images easily. In addition, while the researcher used pain care terminology according to the guidelines, the family members and researchers attached illustrations to the questionnaire. Following the instructions, participants were asked to recall and answer questions about their experiences with their youngest infant hospitalized in the NICU and the lowest birth weight infant hospitalized in the NICU on the same birth date in the case of multiple births.

### 2.6 Data analysis

Descriptive statistics such as the mean, median, and standard deviation (range) were calculated for the attributes using IBM SPSS Statistics Ver.29. Several researchers interpreted and coded the core text of the descriptions, and similar codes were organized into categories to ensure reliability. Additional medical professionals supervised the analysis to ensure its validity in the context of NICUs.

### 2.7 Ethical considerations

The Ethics Committee of Osaka Metropolitan University Graduate School of Nursing approved this study (approval number: 2023-51).

The researchers clearly stated in the request documents that they would respect any decision made by the recruiting institutions and each approached parent, whether or not to join the present research, that parents would have no disadvantage if they declined to participate in the study, that there would be no compensation for participating in the study, that participants would be responsible for communication costs related to the web survey, and that security measures would prevent information leaks.

The anonymous survey began with a question asking participants to provide their consent to participate in the study. Additionally, the researchers obtained their consent separately to provide sensitive personal information, such as the gestational age of birth and weight of the infant. Before submitting their responses, participants were reminded that they could stop answering the survey or refuse to participate even after they had started it but could not delete their responses after they had been submitted.

## 3 Results

Of the 110 people who consented to participate in the survey, two did not complete the questionnaires. Thus, 105 mothers (97.2%) and three fathers (2.8%) were included in the final analysis. Of these 108 participants, two did not consent to provide information about their infants, resulting in 106 participants when analyzing infant-related information.

### 3.1 Demographic data

Table 1 shows the attributes of parents and their infants hospitalized in the NICU between 2015 and 2019 (the first half of the survey period, after the first edition of the guidelines had been published) and between 2020 and 2024 (the second half of the survey period, after the publication of the second edition of the guidelines), with 54 participants (50.0%) in each period. The most frequent attributes of parental visits were more than five visits per week (*N* = 66, 65.7%) and <1 h in the NICU per visit (*N* = 45, 41.7%). As shown in Table 2, the responses covered all regions. The most common attributes of neonates at birth were gestational age <28 weeks (*N* = 61, 57.5%) and birth weight <1,000 g (*N* = 66, 61.1%).

**Table 1:** Demographic data (parents).

	*N*	Rate (%)
Respondent (*N* = 108)		
Mother	105	97.2
Father	3	2.8
Family members accompanying the child (*N* = 108)		
None (respondents only)	17	15.7
Partner (mother/father)	18	16.7
Grandparents/stepparents	1	0.9
Number of fetuses during pregnancy (*N* = 108)		
1	97	89.8
2	11	10.2
Year of birth (*N* = 108)		
2015–2019	54	50.0
2020–2024	54	50.0
Number of visits per week (*N* = 108)		
1-2	22	20.4
3-4	15	13.9
5-6	16	14.8
7	55	50.9
Visiting time per week (*N* = 108)		
<1 h	45	41.7
<15 min	6	5.6
15–30 min	16	14.8
30–60 min	23	21.3
1–2 h	23	21.3
2–3 h	16	14.8
≥3 h	24	22.2

**Table 2:** Demographic data (infants).

	*N*	Rate (%)
Gestational age at birth (*N* = 106)		
<24 weeks	19	17.9
24–26 weeks	26	24.5
26–28 weeks	16	15.1
28–30 weeks	12	11.3
30–36 weeks	28	26.4
37–42 weeks	5	4.7
Birth weight (*N* = 106)		
<500 g	16	15.1
500–750 g	32	30.2
750–1000 g	18	17.0
1000–1500 g	17	16.0
1500–2000 g	17	16.0
2000–3000 g	6	5.7
Medical treatment and environment (*N* = 106)		
Artificial ventilator Tracheal intubation	75	70.8
Nasogastric intubation	91	85.8
Length of hospitalization (*N* = 106)		
<1 month	4	3.8
<2 months	17	16.0
<3 months	19	17.9
<4 months	21	19.8
≥4 months	45	42.5
Regions of hospitalization facilities (*N* = 106)		
Hokkaido and Tohoku	12	11.3
Kanto	42	39.6
Chubu	11	10.4
Kinki	25	23.6
Chugoku and Shikoku	7	6.6
Kyushu	9	8.5

**Figure 1:**
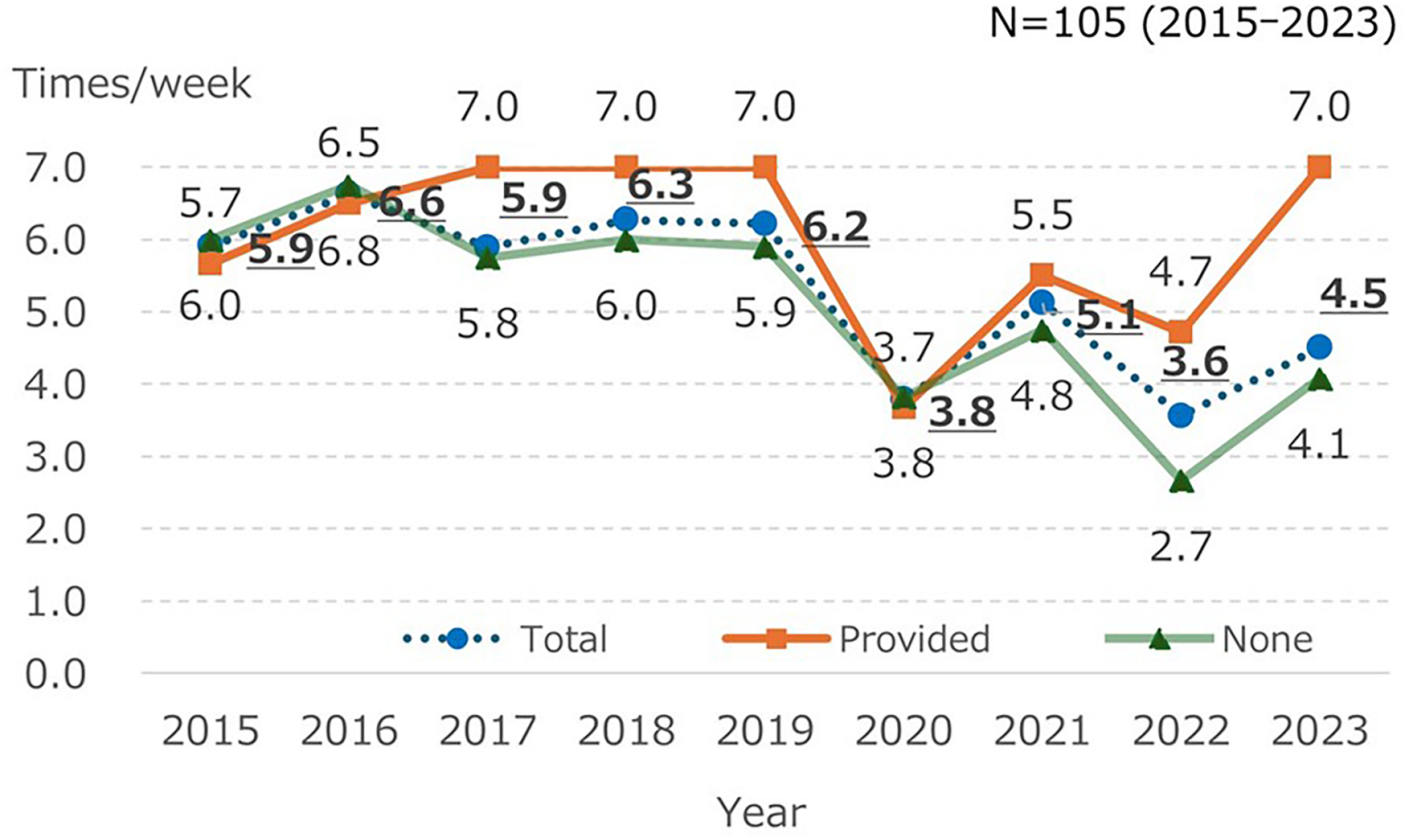
Frequency of visits with and without pain care provided by parents.

In 2020–2022, family visits to the NICU were suspended or restricted due to measures to prevent infection with the novel coronavirus (COVID-19) pandemic, so the frequency of family visits to the NICU decreased (Figure 1). The duration in the NICU per visit was significantly shorter in the category “<1 h” [15.1% vs. 67.3%, respectively; *χ*^2^(3) = 31.210, *p* < .001]. The rate of pain care provided by parents increased over time (45.5% vs. 54.4%, respectively); however, no significant difference was observed [*χ*^2^(3) = 0.249, *p* = .679].

### 3.2 Parental experience with pain care for their infants

**Figure 2:**
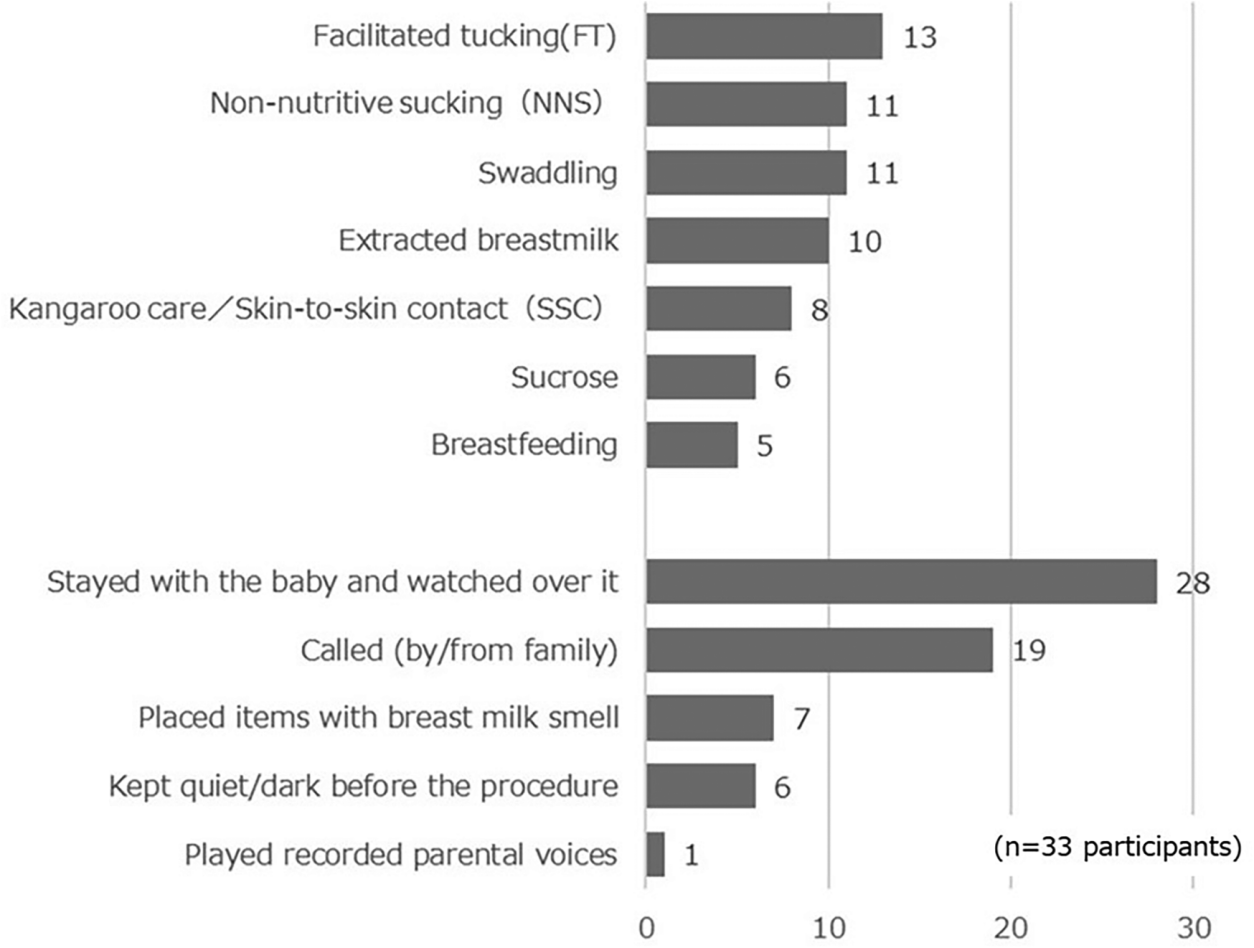
Parental strategies for managing the pain of their infant in the NICU (*n* = 33 participants).

In the parental study population, 33 participants (30.6%) provided pain care to their infants (Figure 2). The most common types of pain care were facilitated tucking (*N* = 13, 39.4%), followed by swaddling and non-nutritive sucking (both *N* = 11, 33.3%), and the use of expressed breast milk (*N* = 10, 30.3%). In addition, the live or recorded voices of a parent (*N* = 20, 62.5%) and smells similar to that of breast milk (*N* = 7, 21%) were used. However, fewer than ten parents used skin-to-skin contact, breastfeeding, or sucrose intake.

### 3.3 Parental intentions to provide pain care for their children

As shown in Table 3, among the participants, 56.5% (*N* = 61) expressed their hope to be involved in further pain care: “I want to do everything I can” and “I would do it if there was an opportunity.” Many parents asked for further support from HCPs (*N* = 44, 40.7%): “I would do it if HCPs recommended it” and “I have never considered accompanying my child or providing pain care.” However, three parents (2.8%), who had provided pain care, did not express an intention to provide further pain care: “I want to continue it to the extent that I usually do” and “I am reluctant (do not want to) accompany the child.”

**Table 3:** Parental intentions to provide pain care to their infants.

		Rate (%)
Intention to participate in pain care for my infant during treatment (*N* = 108)		
Further intentions	61	56.5
I want to do everything I can	47	43.5
I want to do anything Possible	14	13.0
Needs advice	44	40.7
I would try anything if healthcare providers recommend it	13	12.0
I have never considered accompanying my child or providing pain care	31	28.7
No additional support required	3	2.8
I want to continue to do what I usually do	1	0.9
I am reluctant to accompany my child (I do not want to provide pain care)	2	1.9
Reasons for not participating in pain care for their infants (*N* = 75)		
Following the rules (habit of leaving without prompting from medical professionals)	58	77.3
Leaving it to medical professionals	12	16.0
Getting in the way of medical professionals	6	8.0
Thinking there is nothing I can do	3	4.0
Unable to watch it because it is too painful	1	1.3
Others	16	21.3
Treatment not taking place during visiting hours	8	10.7
Request to leave by healthcare provider	4	5.3
Consideration for parents	2	2.7
Visiting restrictions due to COVID-19	2	2.7

As shown in Table 3, the most common reasons for not providing pain care were “following institutional policies (the habit of leaving the room during procedures)” (*N* = 75, 69.4%), “no facilitation of pain care from HCPs” (*N* = 58, 77.3%), “leaving it to the HCPs” (*N* = 12, 16.0%), and “being a nuisance to medical professionals” (*N* = 6, 8.0%). Ten parents evaluated themselves as unhelpful toward their infants or HCPs.

### 3.4 Parental intentions to provide individual recommended pain care methods

As shown in Table 4, of the 100 parents (92.6%) who specified the pain care they would like to provide, Swaddling, Facilitated tucking, and Skin-to-skin contact were the most popular options (60%≤). However, sucrose feeding and breastfeeding (13.0%, respectively), expressed breast milk, and non-nutrition sucking (3.7%, respectively) were less frequently chosen for pain management in the NICU. However, 31 parents (28.7%) specified the pain care methods that they were hesitant to provide, with the most mentioned methods being the use of sucrose and expressed breast milk (both *N* = 14, 13.0%), followed by skin-to-skin contact (*N* = 7, 6.5%), and the use of expressed breast milk and non-nutritive sucking (both *N* = 4, 3.7%).

**Table 4:** Parental requests for recommended pain care (*N* = 108).

	Hope		Hesitation	
	*N*	Rate (%)	*N*	Rate (%)
Swaddling	77	71.3	0	0.0
Facilitated tucking	76	70.4	0	0.0
Skin-to-skin contact	66	61.1	7	6.5
Expressed breast milk	63	58.3	4	3.7
Non-nutritive sucking	54	50.0	4	3.7
Breastfeeding	51	47.2	14	13.0
Sucrose	39	36.1	14	13.0
N/A	8	7.4	77	71.3
N/A: not applicable				

### 3.5 Specific reasons why parents decline their involvement in pain care

As shown in Table 5, the reasons for hesitating to implement the recommended pain care strategies included a lack of knowledge about general pain care for preterm infants, discrepancies with parental values, impracticability due to the infant's physical condition, and breastfeeding difficulties for the mother or child.

**Table 5:** Specific reasons why parents would have declined pain care.

Categories	
Code	Description
Lack of knowledge about pain care leads to indifference and doubt	
No apparent reason	There is no apparent reason. None
Out of hand	I thought it would be counterproductive if there was a possibility that I would not be able to assume a safe position. I felt it would be dangerous if my infant moved. I thought it would not be accessible if I moved. I was scared
Not considered because of potential alternatives	There are several ways to ease the pain, and I do not want her to consume sucrose. Breast milk is better. I was producing breast milk, so I tried to use that
Lack of knowledge	I do not know whether sucrose harms my baby if it is safe for babies if I can see the evidence that it suits my baby. I did not understand enough
Sudden invitation to participate in care	I think I would be confused if I was suddenly invited. If I could understand it with a prior explanation, I might want to participate
Discrepancy between recommended pain care method and parental values	
Prioritizing child-rearing views	I did not want my child to use a pacifier. I do not breastfeed or provide kangaroo care, even though they recommend it, because I feel like it takes away a mother's happy and healing time. I wanted to do it in a way only a parent can do
Concerns associated with pain	I do not want my child to think that breastfeeding hurts. I am worried that the care I give them will leave a painful memory
Reluctance to others seeing your skin	I do not want to be seen if the doctor is a man. Because of discomfort showing my skin/breastfeeding in public. Embarrassed while breastfeeding
Constraints on pain care according to their infants’ circumstances	
Instability of the child's overall condition	The baby had no sucking power. Because my child was sick, he could not eat sweets
Difficulties with breastfeeding	Breastfeeding was not going well. Because she got used to the bottle and could not breastfeed. Even when I was breastfeeding, she would forget to breathe and develop cyanosis, so I was worried about breastfeeding
Hesitation about the burden on children	I am also afraid of my child's aspirations. Breastfeeding seemed like it was a burden for my child. I am worried that my baby will choke on breast milk when he is crying

## 4 Discussion

We obtained valuable data about parental intentions and requests to provide pain care for their infants through experiential reflections from 108 parents, including 66 parents (62.3%) with extremely low birth weight infants weighing less than 1,000 g at birth regarding pain care. Parents expressed that they would like to have more opportunities and receive more advice regarding recommended pain care methods than experienced when their child was treated at a NICU. Reasons for hesitation to implement recommended pain management practices included a lack of knowledge about general pain care for preterm infants, discrepancies with parental values, impracticability due to the infant's physical condition, and feeding difficulties for the mother or child. These new findings are essential for NICU staff to improve parental involvement in pain management during procedures.

As shown in Figure 2, parents rarely used skin-to-skin contact, expressed breast milk, or sucrose for pain management in their children. A survey among Japanese HCPs in 2021 also reported that skin-to-skin contact, breast milk, and sucrose were used by less than 30%. Cruz et al. (9), a synthesis of epidemiological surveys, also reported that breast milk and kangaroo care are rarely used, whereas sucrose is often used in many countries. Thus, while domestic and international findings regarding breast milk and kangaroo care were consistent, sucrose trends differed.

Concerns were reported that visiting restrictions from 2020 to 2023 to prevent the spread of COVID-19 may impact skin-to-skin contact and breastfeeding. Ozawa et al. (7) reported that during the COVID-19 pandemic, the practice of kangaroo care and breast milk expression in Japanese NICUs has declined. Thus, parents may have had fewer opportunities to participate in pain care due to the COVID-19 pandemic. However, the major reason that parents had no experience with pain care for their infants was the presence of institutional rules, e.g., the habit of leaving the infant who undergoes a procedure, unless prompted by medical staff). Some parents seemed worried about their confusion and viewed the responses of the facility favorably. Yokoo and Ozawa (3) also reported that reasons of parents for not wanting to accompany their children included that they wanted to follow the rules, trusted the HCPs, wanted to prevent emotional upset, and were concerned about the future relationship with medical staff. McNair et al. (10) reported that HCPs, as gatekeepers, influenced parental commitment during NICU procedures. HCPs are inconsistently able to assess parental readiness (11, 12), and the knowledge of HCPs, their familiarity with pain management strategies, and their ability to communicate with family members considerably affects parental participation (13).

Our survey results also show that in Japan, HCPs function as gatekeepers. Some HCPs understood the situation of the parents and their infants and considered the parental provision of pain care. In other cases, when HCPs do not facilitate pain care because they assess the parents not to be prepared for their involvement, parents may perceive that they are not allowed to do so due to facility rules. Therefore, we propose that HCPs reassure parents repeatedly that they are allowed to alleviate their infants' procedural pain and that parents should freely share their anxieties, worries, wishes, and requests with HCPs so that they proceed at the parents' pace.

Regarding the intentions of accompanying their infant during procedures and being involved in pain management, the participants of our study expressed both a desire for more opportunities and the need for more support from HCPs. Parental requests for future pain care strategies were more frequent for skin-to-skin contact and the use of expressed breast milk, which had low implementation rates, than for non-nutritive sucking and breastfeeding.

Parents need support from HCPs to participate in pain care but expect HCPs to guide them (11, 14, 15). McNair et al. (10) reported in a meta-synthesis that parents are aware of how to interact with their hospitalized children under various conditions of stress and anxiety and that knowing how to interact with them also influences parental involvement. Similarly, Yokoo et al. (3) reported that to protect their children from pain, parents wanted to be able to recognize pain signs and implement pain relief methods themselves, and they hoped that HCPs would provide pain care based on the latest evidence. Therefore, HCPs should routinely state that they will alleviate the infants' pain during procedures; that parents can freely share their fears, worries, hopes, and requests with HCPs; and that the HCPs care for the infant at a pace comfortable for the parents.

Although recommended, parents are reluctant to use skin-to-skin contact, breastfeeding, and the use of expressed breast milk or sucrose as pain management strategies. Sucrose was the least desired option, and many respondents did not want to use it. Factors influencing the selection of pain care methods included the lack of knowledge about general pain care for premature infants, discrepancies with parental values, constraints due to the infant's physical condition, and breastfeeding difficulties for the mother or child. In their meta-synthesis, McNair et al. (10) stated that the comfort of the NICU environment also influences parents who are stressed and anxious. For parents to provide pain care to their children, families are concerned not only with the pain care provided by medical professionals but also with safety aspects, the child's physical condition, sucrose intake, breastfeeding status, and the burden on the mother and child during skin-to-skin contact. Therefore, we propose that HCPs confirm parental wishes and requests regarding pain care and provide a support system that includes providing a comfortable NICU environment where concerns and questions can be discussed so that care can be provided to the child without the parents feeling threatened.

We would like to suggest standardization of care based on screening the general postpartum depression assessment tool in the NICU. The Ministry of Health Welfare Labor, Japan Society of Obstetrics and Gynecology, and the Japan Association of Obstetricians recommend that screenings for all postpartum women, such as “The Edinburgh Postnatal Depression Scale” in obstetric departments and community (16, 17), however, not routinely shared with the NICU unless severe depression symptom. NICU HCPs try to consider individualized plans for their involvement and implement them for their infants based on the parent's mental health status totally by talking to the parents to check for any stress factors, which might be hard work. Therefore, there is an urgent need for HCPs to standardize systematic care interventions with standardized screening to increase the likelihood of seeking involvement, increase anxiety, and provide reassurance to infants in the NICU.

This study has several limitations. First, recall bias may exist because parents were surveyed after their children had been discharged from the NICU. This strategy was chosen because of concerns that asking parents about their infant's pain during hospitalization might increase the psychological burden on the family. Second, despite the use of illustrations and explanations, parents may experience confirmation bias in intuitively associating their own experiences with pain care because disparities in the prevalence of pain care across facilities exist. Third, selection bias might exist owing to the inclusion of those parents who were more interested in implementation of pain care for their preterm infants in the NICU, actively collected information about childcare, interacted with peers, and were highly interested in the role of parents in the care of their children. Therefore, compared with the responses of parents whose children are hospitalized in a general NICU, the descriptions of their intentions and requests regarding pain care may be constructively reflected, which is a limitation of this study. However, in this survey, we believe that parents shared their wishes and requests regarding pain care with the HCPs. It is necessary to further clarify the issues faced by HCPs in Japan to improve the quality of care for parents and their infants.

## 5 Conclusion

Our findings elucidate the intentions and requests of parents regarding their infants' pain care. The parents also provided specific reasons for hesitating to implement individual pain care methods. This survey demonstrated that when parents provide pain care for their children in the NICU, they are requested to make choices based on advice and knowledge offered by HCPs, taking into account the parents' diverse values as well as the overall condition of their infant and their breastfeeding status. Therefore, we suggest that HCPs support parents in choosing not only the recommended care but also the most appropriate pain care for the condition of their infant.

## Data Availability

The raw data supporting the conclusions of this article will be made available by the authors, without undue reservation.
